# Clinical Value of Susceptibility Weighted Imaging of Brain Metastases

**DOI:** 10.3389/fneur.2020.00055

**Published:** 2020-02-04

**Authors:** Daniel Schwarz, Martin Bendszus, Michael O. Breckwoldt

**Affiliations:** ^1^Neuroradiology Department, University Hospital Heidelberg, Heidelberg, Germany; ^2^Clinical Cooperation Unit Neuroimmunology and Brain Tumor Immunology, German Cancer Research Center (DKFZ), Heidelberg, Germany

**Keywords:** MRI, brain metastases, SWI, treatment monitoring, radiotherapy

## Abstract

MRI is used for screening, initial diagnosis and follow-up of brain metastases. Multiparametric MRI protocols encompass an array of image sequences to depict key aspects of metastases morphology and biology. Given the recent safety concerns of Gd-administration and the retention of linear Gd-agents in the brain, non-contrast sequences are currently evaluated regarding their diagnostic value for brain imaging studies. Susceptibility weighted imaging has been established as a valuable clinical and research tool that is heavily used in clinical practice and utilized in diverse pathologies ranging from neuroinflammation, neurovascular disease to neurooncology. We review the value of SWI in the field of brain metastases with an emphasis on its role in early diagnosis, determination of the primary tumor entity, treatment monitoring and discuss therapy-associated changes that can affect SWI. We also review recent insights on the role of “isolated SWI signals” and the controversy on the specificity of SWI for the early detection of brain metastases.

## Introduction

Brain metastases (BM) are highly relevant in solid cancer patients and contribute significantly to overall morbidity and mortality (1). MR imaging is the gold standard for early diagnosis and treatment monitoring of BM patients (2). Treatment of BM is mainly based on neurosurgical resection and radiotherapy with a limited role for systemic chemotherapy due to low efficacy (3, 4). Recent immunotherapeutic trials have shown promising results in a subset of BM patients and are currently tested in clinical practice (5, 6). All such therapeutic regimes require regular and standardized MRI follow-up for disease monitoring to detect changes in the tumor micromilieu (TME) that occur during therapy.

In general, brain metastases imaging has three major goals: (a) early detection, (b) determination of the primary tumor entity, and (c) tumor monitoring, including differentiation between tumor progression and treatment related effects. Susceptibility weighted imaging (SWI) can contribute to all three challenges and this review will highlight these different aspects. SWI has been first described in 1997 when it was introduced for venous imaging (7). After the original description SWI has been widely used in clinical and preclinical studies (8–10). In clinical practice SWI is used for the detection of iron, hemorrhage and microbleedings (11–14) but has also been widely investigated in the field of neurovascular disease (15), for clot detection in stroke (16), in neurooncology (17, 18), neurotrauma (19), and autoimmune disease (20–23). Also neurosurgical applications of SWI have been recently reviewed (24).

## Origin of the SWI Contrast

The SWI signal originates from varying intrinsic susceptibilities that are present between voxels and get out of phase at longer echo times, leading to signal loss in the respective voxel. Susceptibility is altered by paramagnetic and diamagnetic materials such as deoxygenized hemoglobin within veins, tissue calcifications or iron depositions. Susceptibility is further introduced through the distortion of the magnetic field, e.g. at tissue boundaries or by metal implants. A thorough derivation of the physics behind SWI is beyond the scope of this article and has been covered by previous reviews (25).

In brief, for the generation of SWI, phase images are high-pass-filtered and transformed to a phase mask which is then multiplied on the magnitude image to increase contrast (25). It is important to note that the resulting image contrast depends on the manufacturer and the post-processing used: In a right-handed system, paramagnetic phase signals (like hemosiderin and deoxyhemoglobin) are depicted as dark voxels while diamagnetic phase signals (like calcifications) are shown as bright voxels. In a left-handed system, the images produced follow the inverse greyscale.

Newer developments in SWI include quantitative susceptibility mapping (QSM) and susceptibility tensor imaging which allow the quantitative measurement of the susceptibility in a given voxel (26–31). Recently, the minimum size of histo-pathologically confirmed microhemorrhages that can be depicted by clinical SWI were established (32). In this work, MR-positive microbleeds were typically found to correspond to histopathological hemorrhages of 3.6 mm^3^ whereas MR false-negative microbleeds were found to be significantly smaller in size with an average volume of 0.3 mm3 on histopathology.

## SWI for Brain Tumor Imaging

In the brain tumor field SWI has been recently reviewed for glioma imaging (17). It is important to note that recent studies have shown that SWI can aid in glioma grading because of its sensitivity for (micro-)hemorrhages and the microvasculature itself that correlates with tumor grade (33). In particular, several authors found that the amount and extent of SWI artifacts correlated well with the grading of gliomas with more artifacts being correlated to higher tumor grade and increased neoangiogenesis (34–36). This could further be confirmed by quantification of intratumoral SWI patterns using fractal image analysis (37).

For monitoring of brain metastases multiparametric anatomical imaging is performed in the routine clinical setting (38). Further advanced sequences including chemical exchange saturation transfer imaging (CEST), magnetization transfer (MT) imaging and MR spectroscopy (MRS) have also been assessed regarding their clinical value and have recently been reviewed (39). In the context of neurooncology MT imaging could differentiate glioblastoma from brain metastasis (40). Interestingly, magnetization transfer imaging showed subtle changes also in the normal appearing white matter of the contralateral site that did not show obvious changes on standard MRI sequences (41), indicating that MT imaging might be more sensitive to detect subtle, tumor-induced changes. CEST was shown to enable the detection of radiotherapy induced apoptosis (42, 43). MRS has been used to differentiate radiation necrosis from tumor progression albeit with limited specificity (44).

## SWI for Differentiation of the Underlying Tumor Entity

As SWI provides an image contrast that is different from conventional spin echo MR sequences, the susceptibility information can reveal additional features of the tumor microenvironment. The concept of “intratumoral susceptibility signals” (ITSS) was introduced as a semiquantitative parameter that is comprised of “low-signal tubular structures or dot-like structures with or without conglomeration within a tumor” (45) that are indicative of tumor microbleedings or neovessels and indicate highly malignant lesions.

Using this approach, it was shown that metastases could be differentiated from GBM due to higher ITSS numbers in GBM, as well as high-grade gliomas from lymphomas and non-tumorous brain lesions (46).

**Figure 1:**
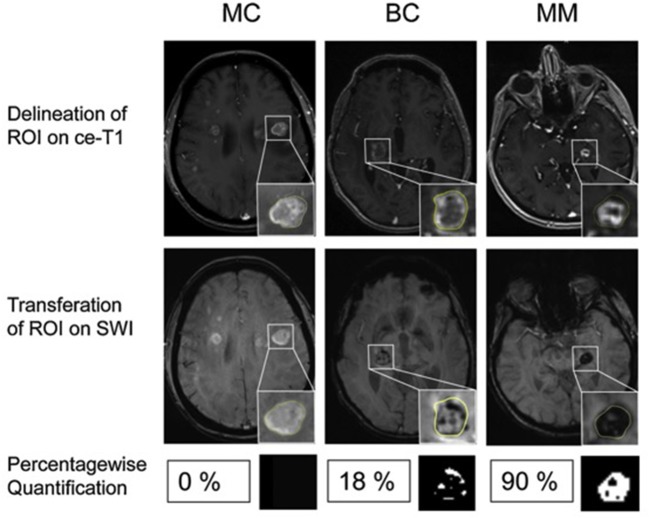
Differentiation of brain metastases by ITSS. Examples of patients with MC, BC and MM. **(Upper)** Contrast enhanced T1-weighted images (ce-T1). **(Middle)** Contrast enhanced susceptibility weighted images (SWI). Insets: Delineation of the enhancing lesion on ce-T1 images and corresponding ROI on SWI. **(Lower)** Percentagewise ITSS quantification with corresponding binarized ITSS map. No ITSS (0%) in MC, minor ITSS (18%) in BC and subtotal ITSS (90%) in MM. Adapted from Radbruch et al. (48).

However, the exact grading-scheme remained relatively reader-subjective, so further efforts were made subsequently to achieve a more objective, less reader-dependent measure. Percentage-wise quantification of ITSS using binarized mask of the SWI map compared the three most common metastatic entities in the brain, namely bronchial carcinoma (BC), mamma carcinoma (MC), and malignant melanoma (MM) (47) (Figure 1). This approach could discriminate MM from MC [area under the receiver operating characteristic curve (AUC) of 0.96] or BC (AUC of 0.81) while there was no clear cut-off between MC and BC. Specifically, only 1/20 MC patients showed more than 8% ITSS in contrast to 10/15 patients with MM. This indicates that different brain metastatic entities have different growth behavior, neoangiogenesis induction and aggressiveness, which can be inferred by SWI.

An important addition to this observation was made by Franceschi et al. who reported a correlation of ITSS and metastatic size (49): while micrometastases (i.e., <0.1 cm3) only rarely showed ITSS (10/342), ITSS drastically increased in macrometastases (i.e., >0.1 cm3, in 410/610 metastases). In this latter subpopulation, a higher propensity of ITSS in MM compared to MC was confirmed (76.9 vs. 55.6%).

## SWI for Initial Diagnosis of Brain Metastases

While SWI thus appears as a promising imaging contrast to contribute to the determination of the primary tumor entity in BM, it remained controversial if SWI is helpful for initial, early diagnosis of BM. This seems even more important in light of the recent discussions on the significance of gadolinium depositions in brain tissue following the exposure to gadolinium-containing contrast agents (50). This question was addressed in a recent work on the diagnostic performance of different MR sequences in the early detection of melanoma brain metastases (38): In this work on a large retrospective cohort of more than 1200 patients, diagnostic sensitivity was compared between six different MR sequences, including SWI. The authors found that SWI did not reach the diagnostic sensitivity of contrast-enhanced T1-weighted imaging (64.7 vs. 99.7%). Interestingly, SWI also showed a lower sensitivity compared to FLAIR imaging (77.0%) but could outperform T2-weighted imaging (61.0%), non-contrast enhanced T1-weighted imaging (56.7%) and DWI (48.4%).

While data on other brain metastatic entities is currently lacking, it appears reasonable to assume that current SWI will not replace contrast-enhanced T1-weighted imaging for the early detection of metastatic brain disease because the underlying effects, namely the accumulation of paramagnetic ions and microbleedings, appear later than the early disruption of the blood brain barrier—which is delineated by ce-T1w-imaging.

## SWI in Melanoma Brain Metastases

**Figure 2:**
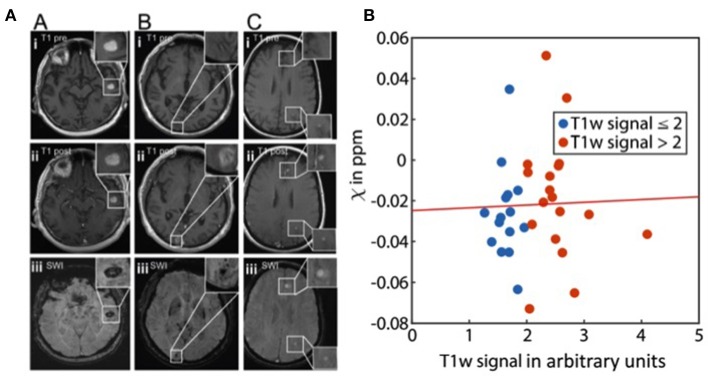
Melanoma metastases and susceptibility. **(A)** Imaging findings in melanotic and amelanotic brain metastases. T1 pre-Gd-contrast (i), post-Gd-contrast administration (ii), and SWI (iii) images are shown for melanotic (A) and amelanotic (B) brain metastasis. (C) Examples of metastases with melanotic and amelanotic imaging features in a single patient. Adapted from Schwarz et al. (54). **(B)** Scatter plot showing the relation of susceptibility values (χ) and normalized T1w signal of melanoma metastases (*p* = 0.87). The line represents a linear fit. From Straub et al. (55).

Among malignant entities to metastasize to the brain, malignant melanoma plays a special role with regard to susceptibility effects. While SWI signal loss can relatively easily be attributed to (micro-)hemorrhage in other entities, melanin itself in MM may lead to susceptibility effects due to paramagnetic metal scavenging which is known to cause non-contrast-enhanced T1w-hyperintensity (51). This would imply that susceptibility-related signal losses could potentially indicate metastatic lesions which are not detectable in standard sequences. This was first reported by Gaviani et al. on T2^*^-weighted imaging in three malignant melanoma patients (52). However, later studies analyzing the fate of isolated cerebral SWI artifacts in larger patient cohorts over time could not confirm the hypothesis that such “isolated SWI signals” would eventually evolve into overt brain metastases (53, 54). Indeed, these studies showed that SWI signal losses without corresponding signal changes on standard sequences remained constant over time. On the other hand, it was reported that T1w-hyperintense melanotic metastases did not exhibit a higher frequency of SWI signal losses as compared to amelanotic metastases and the radiological presentation between cases could vary considerably (Figure 2A). Additionally, in another recent study significant differences in the susceptibility between melanotic and amelanotic brain metastases as measured by QSM could also not be demonstrated; nor could a correlation to T1-weighted signals be found, further underpinning that melanin *per se* does not account for a detectable paramagnetic effect *in vivo* (55) (Figure 2B).

## SWI in the Assessment of Treatment Responses

**Figure 3:**
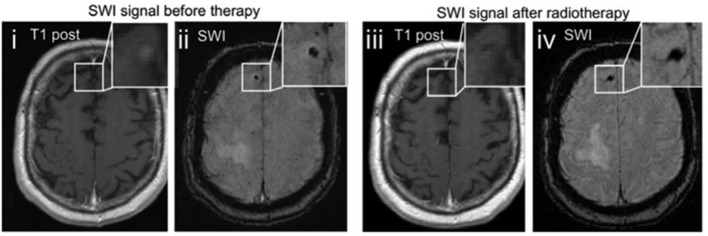
Therapy related changes of SWI. Example images of a melanoma metastasis before (i, ii) and after radiotherapy (iii, iv). The punctuate contrast enhancement in the right frontal lobe (i) disappears after stereotactic radiotherapy (iii), being consistent with radiological remission. The SWI signal drop remains as a remnant of the preexisting metastasis (iv). Adapted from Schwarz et al. (54).

Interestingly, Schwarz et al. found a significantly higher prevalence of isolated SWI artifacts among patients with brain metastases as compared to melanoma patients without metastatic brain disease (54). Of those patients, only patients after radiotherapy showed an increased number of such artifacts indicating that these findings did not constitute vital tumor tissue but may rather represent either non-specific microbleedings or radiotherapy-related parenchymal damage (56) which are both well-known phenomena in patients after radiotherapy of the brain (57). As a third explanation of these findings the authors proposed the possibility of posttherapeutic remnants of former metastatic lesions because in cases of radiological remission of treated metastases, only an isolated SWI artifact persisted (Figure 3).

As many patients suffering from BM either receive stereotactic or whole-brain radiotherapy, it is crucial during tumor monitoring to differentiate between “pseudoprogression” following successful treatment and true recurrence of the disease (58, 59). Although not applied to brain metastases so far, R2^*^-mapping, another susceptibility-related imaging approach (60) has recently been introduced as a promising imaging marker to differentiate pseudoprogression from progressive disease in glioblastoma multiforme (61). The authors reported a rim of high R2^*^ values with an accompanied SWI-hypointensity as indicative of pseudoprogression as well as a ratio of R2^*^ in the contrast-enhancing to the non-contrast enhancing lesion close to 1. Conversely, a ratio of >1.3 was found in patients with true progression. According to this quantification a correct diagnosis was achieved in 9/9 patients. Similarly, promising results were reported in a preclinical model by the same group (62).

## Potential Future Applications: Texture Analysis and Radiomics

A number of methods have recently been introduced to extract multiple image features from MRI data to create high dimensional signatures of a given tumor. Such features of varying complexity can then, via a dedicated model, be used to predict certain target variables, in most cases histopathological or clinical parameters possibly having an impact on treatment decisions and prognosis. This multi-step process is broadly referred to as “Radiomics” (63–65).

Expanding the input parameter space by adding complementary contrast with new information may provide new features and lead to a higher classification accuracy and reliability. SWI has just started to be incorporated into such models proving that it can indeed provide complementary discriminators, e.g., in the differentiation of glioblastoma and solitary brain metastases (66). It needs to be determined in future studies to which extent SWI will play a role for these applications.

## Summary and Outlook

SWI is a valuable image sequence that utilizes phase information to produce an image contrast different from standard anatomical MR sequences. It therefore provides complementary tissue information to further characterize brain lesions like brain metastases. While it does not appear to be usable as a sole image modality in metastatic brain disease lacking sensitivity and specificity, it can contribute important supplementary information on the underlying tumor entity and during treatment monitoring. In the future, quantitative susceptibility mapping may further refine tumor MR signatures, which could be used in texture and radiomic analysis to non-invasively support early detection and treatment monitoring of metastatic brain disease.

## Author Contributions

All authors listed have made a substantial, direct and intellectual contribution to the work, and approved it for publication.

### Conflict of Interest

The authors declare that the research was conducted in the absence of any commercial or financial relationships that could be construed as a potential conflict of interest.
